# Convergent functional changes of the episodic memory impairment in mild cognitive impairment: An ALE meta-analysis

**DOI:** 10.3389/fnagi.2022.919859

**Published:** 2022-07-15

**Authors:** Xuhong Liang, Qianqian Yuan, Chen Xue, Wenzhang Qi, Honglin Ge, Zheng Yan, Shanshan Chen, Yu Song, Huimin Wu, Chaoyong Xiao, Jiu Chen

**Affiliations:** ^1^Department of Radiology, The Affiliated Brain Hospital of Nanjing Medical University, Nanjing, China; ^2^Institute of Brain Functional Imaging, Nanjing Medical University, Nanjing, China; ^3^Department of Neurology, The Affiliated Brain Hospital of Nanjing Medical University, Nanjing, China; ^4^Institute of Neuropsychiatry, The Affiliated Brain Hospital of Nanjing Medical University, Nanjing, China

**Keywords:** resting state, episodic memory, activation likelihood estimation, functional connectivity, regional homogeneity, the amplitude of low-frequency fluctuation, mild cognitive impairment

## Abstract

**Background:**

Mild cognitive impairment (MCI) is considered to be an intermediate stage between normal aging and Alzheimer's disease (AD). The earliest and most common symptom of MCI is impaired episodic memory. When episodic memory is impaired in MCI patients, specific functional changes occur in related brain areas. However, there is currently a lack of a unified conclusion on this change. Therefore, the purpose of this meta-analysis is to find MRI-specific functional changes in episodic memory in MCI patients.

**Methods:**

Based on three commonly used indicators of brain function: functional connectivity (FC), the amplitude of low-frequency fluctuation /fractional amplitude of low-frequency fluctuation (ALFF/fALFF), and regional homogeneity (ReHo), we systematically searched PubMed, Web of Science and Ovid related literature and conducted the strict screening. Then we use the activation likelihood estimation (ALE) algorithm to perform the coordinate-based meta-analysis.

**Results:**

Through strict screening, this meta-analysis finally included 21 related functional neuroimaging research articles. The final result displays that functional changes of episodic memory in MCI patients are mainly located in the parahippocampal gyrus, precuneus, posterior cingulate gyrus, cuneus, middle temporal gyrus, middle frontal gyrus, lingual gyrus, and thalamus.

**Conclusions:**

There are specific functional changes in episodic memory brain regions in MCI patients, and the brain functional network can regulate episodic memory through these brain regions. And these specific changes can assist in the early diagnosis of MCI, providing new ideas and directions for early identification and intervention in the process of MCI.

## Introduction

Mild cognitive impairment (MCI) is recognized as an intermediate condition of cognitive alteration between normal aging and Alzheimer's disease (AD) (Kim et al., 2020). The prevalence of MCI in the elderly over 65 is 15% to 20%, 15% of which will progress to dementia at different times thereafter (Kim et al., 2020). And the average annual conversion rate is higher than that of the general population (Petersen et al., 2001). The earliest and most common clinical symptoms of AD are episodic memory loss, accelerated forgetting, and impaired delayed memory (Aurtenetxe et al., 2016). Numerous resting-state functional magnetic resonance imaging (rs-fMRI) studies have shown that there are abnormalities in brain regions and large-scale brain functional networks in MCI and AD patients (Yuan et al., 2016). Episodic memory is a complex physiological activity involving multiple brain regions and networks (Bai et al., 2009a). Therefore, the changes of brain regions related to episodic memory are closely related to the progression of AD. We aimed to analyze the results of functional changes of episodic memory to find radiographic markers of impaired episodic memory in MCI.

Episodic memory is the ability to retain, recall, and encode information about events and experiences concerning a specific time and place (Bai et al., 2016). There are many studies on episodic memory, and the main brain regions involved in episodic memory are the hippocampus, parahippocampal gyrus, entorhinal cortex, posterior cingulate gyrus, precuneus, cuneus, middle frontal gyrus, middle temporal gyrus, and lingual gyrus (Gliebus, 2018; Shi et al., 2020). A functional connectivity study by Bai et al. (2009a) confirmed that the process of episodic memory relies on a highly interconnected neural circuit and involves multiple brain networks and regions. Overall decline in episodic memory retrieval or recognition is a central feature of MCI and is closely related to disease progression (Wolk et al., 2013; Dubois et al., 2014). However, at present, there is no unified standard for the research on the changes of episodic memory function, and it is difficult for imaging to assist diagnosis. Thus, the research on the changes in episodic memory function is crucial.

Rs-fMRI, with its high temporal resolution and non-invasive features, has been widely used to study functional brain activity and cognitive deficits in AD and MCI (Yan et al., 2019). There are three indicators commonly used to study brain function: functional connectivity (FC), the amplitude of low-frequency fluctuation /fractional amplitude of low-frequency fluctuation (ALFF/fALFF), and regional homogeneity (ReHo) (Cheng et al., 2019). FC measures the dependent internal fluctuations of blood oxygenation level-dependent (BOLD) in different brain regions, analyzes the cooperation of different brain regions at rest or performing tasks, and also provides temporal correlations between physiological activities (Zhu et al., 2021). ALFF/fALFF reflects neural activity from the perspective of brain energy, which is of great significance in the study of brain network function (Wang et al., 2019). ReHo can reflect the consistency of spontaneous neuronal activity in the brain and is a functional indicator that can effectively distinguish normal elderly people from MCI patients (Liu et al., 2021). These three approaches, from different perspectives, provide a more comprehensive understanding of the changes in brain function in normal older adults and MCI. The biomarkers found through these three brain function indicators can detect changes in brain function before neuronal damage becomes irreversible, and play an important role in intervening in the disease process.

Activation likelihood estimation (ALE) technique is a meta-analysis method that calculates the consistency of statistically important focal points in experiments based on the probability distribution centered on the coordinates of each set of focal points (Costa et al., 2021). This approach has significant implications for studies with inconsistent findings because it can theoretically analyze the most stable changes in brain activity (Goodkind et al., 2015). Rs-fMRI is a functional imaging technique that requires participants to rest throughout the whole process and reflects the underlying neuronal activation patterns in brain regions (Noroozi and Rezghi, 2020). Therefore, rs-fMRI has been widely used to analyze functional differences between MCI patients and cognitively normal elderly people (Cha et al., 2015). In this study, the ALE technique was used to study the functional changes of episodic memory-related brain regions in MCI during resting state, and to find the characteristics of early MCI brain functional changes.

Hence, the purpose of this study is to summarize and evaluate the specific changes of episodic memory-related brain regions in MCI patients and to discuss its association with brain networks and its significance for early diagnosis of MCI patients. We hypothesized that (1) there would be specific changes in FC, ALFF, and ReHo of episodic memory-related brain regions in MCI patients; (2) The brain network can influence episodic memory through these brain regions. The in-depth summary and understanding of the episodic memory of MCI patients will help to detect the disease earlier and more comprehensively, and to intervene and delay the process of the disease.

## Methods

### Literature search and study selection

The research objectives were systematically studied according to the Preferred Reporting Items for Systematic reviews and Meta-Analyses (PRISMA) and recorded using the suggested checklist.

### Search strategy

We systematically and thoroughly searched PubMed, Web of Science, and Ovid to collect the results of MCI patient function studies. The search keywords are as follows: (1)[(functional magnetic resonance imaging) OR (resting state)] AND (mild cognitive impairment) AND (memory) AND [(Functional connectivity) OR (FC)]; (2) [(functional magnetic resonance imaging) OR (resting state)] AND (mild cognitive impairment) AND [(regional homogeneity) OR (ReHo) OR (local consistency)]; (3) [(functional magnetic resonance imaging) OR (resting state)] AND (mild cognitive impairment) AND [(amplitude of low frequency fluctuations) OR (ALFF) OR (fractional amplitude of low frequency fluctuations) OR (fALFF)].

**Figure 1:**
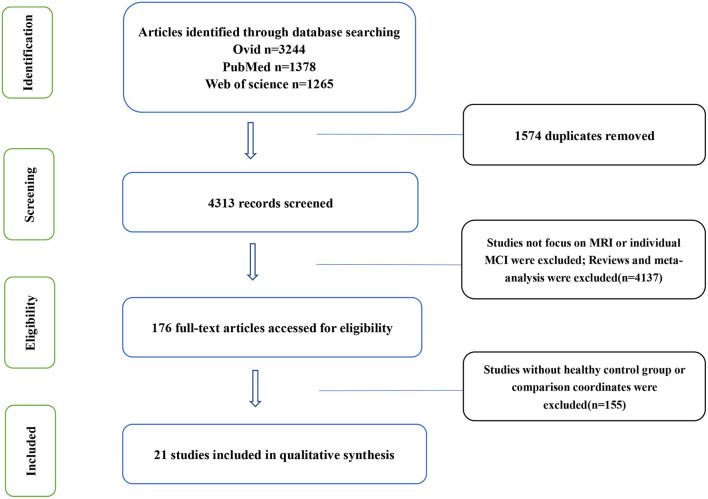
Flow of information through different phases of a systematical review.

According to the listed search formulas, a total of 4,313 articles were retrieved. The literature search and screening steps are shown in Figure 1.

### Inclusion and exclusion criteria

The selected literature must meet the following criteria to be included in this study: (1) Subjects meeting MCI diagnostic criteria; (2) The included studies were performed based on resting states; (3) MCI was compared with the normal control group; (4) Baseline data were included in both the experimental and control groups (5) Coordinate spatial information obtained by Talairach or the Montreal Neurologic Institute (MNI); (6) articles published in peer-reviewed journals;

If any of the following conditions occur, they will be excluded: (1) Subjects with known neurological or psychiatric diseases such as Parkinson's disease, depression, and epilepsy; (2) Subjects suffering from serious diseases, such as cancer, cerebrovascular diseases, and brain trauma; (3) Accepted subjects who have undergone cognitive enhancement training; (4) Secondary research, such as meta-analysis and system review, etc.; (5) Documents where necessary data cannot be obtained.

### Data extraction and quality assessment

Two researchers in the research group independently extracted data and checked the extracted data. First, read each article included in the literature, and then extract the coordinates of abnormal brain function indexes (FC, ALFF/fALFF, and ReHo) related to the episodic memory of patients with MCI. We converted Talairach space into MNI coordinates using the “icbm2tal” transformation, which allowed these foci to be performed in MNI coordinates. During this process, if two researchers disagree, the third researcher will intervene to make a comprehensive judgment.

### Data analysis procedures

To compare the functional changes of MCI and HC, we divided the different results obtained by the three indicators into the increase group and decrease group. MCI: increased FC (251 subjects, 21 foci); decreased FC (191 subjects, 53 foci); increased ALFF (44 subjects, 3 foci); decreased ALFF (62 subjects, 4 foci); increased ReHo (158 subjects, 7 foci); decreased ReHo (250 subjects, 22 foci).

ALE algorithm has experimental consistency and spatial convergence of active focus (Yang et al., 2020). We used Ginger ALE2.3.6 software (http://www.brainmap.org/ale/) to perform ALE calculations. In order to be able to compare the coordinates of the brain regions of different studies, it is first necessary to transform the Talairach coordinates into the MNI space. After that, the extracted information was organized into a text file and imported into the ALE software. We selected the cluster forming threshold at p < 0.01, with 1,000 permutations, and a cluster-level family-wise error (FWE) correction at p < 0.05 to obtain the ALE map.

## Results

### Search results

According to the retrieval formula, 5,887 articles were retrieved from three databases, including 1,574 duplicate articles. After the strict screening, 21 articles were included. There are 9 articles related to FC, including 6 articles with rising FC and 6 articles with decreasing FC. Three articles had both rising and declining results. A total of 303 MCI patients and 309 HCs were included; There are 4 articles related to ALFF, including 3 articles with rising ALFF and 4 articles with decreasing ALFF. Three articles had both rising and declining results. A total of 62 MCI patients and 72 HCs were included; There is no article related to fALFF; There are 8 articles related to ReHo, including 4 articles with rising ReHo and 8 articles with decreasing ReHo. Four articles had both rising and declining results. A total of 250 MCI patients and 260 HCs were included. More detailed information on each of the included studies is shown in Table 1.

**Table 1:** Demographic characteristics of the included rs-fMRI studies.

**References**	**Sample size(n)**	**Age (years** ±**SD)**	**Gender (M/F)**	**MMSE(SD)**	**Group contrasts**	**Foci**	**Threshold**
**FC**							
Chen et al. (2016)	MCI 85	69.48 ± 7.52	46/39	26.25 ± 2.65	MCI > HC	2	*p* < 0.01 cor
	HC 129	68.43 ± 6.59	64/65	28.22 ± 1.43	MCI < HC	16	
Gardini et al. (2015)	MCI 21	70.62 ± 4.66	13/8	/	MCI > HC	3	*p* < 0.05 uncor
	HC 21	69.75 ± 6.45	7/14	/	MCI < HC	0	
Han et al. (2012a)	MCI 40	86.26 ± 4.49	7/33	27.10 ± 1.96	MCI > HC	4	*P* < 0.01 cor
	HC 40	86.28 ± 4.39	15/25	28.68 ± 1.23	MCI < HC	8	
Kang et al. (2021)	MCI 65	77.1 ± 4.2	32/33	21.7 ± 4.4	MCI > HC	3	*P* < 0.01 cor
	HC 37	73.9 ± 2.0	15/22	27.1 ± 1.7	MCI < HC	0	
Li et al. (2018)	MCI 20	66.95 ± 9.65	7/13	24.80 ± 3.70	MCI > HC	0	*p* < 0.05 cor
	HC 20	67.25 ± 7.50	10/10	28.60 ± 1.23	MCI < HC	2	
Bai et al. (2011)	MCI 26	71.4 ± 4.3	19/7	27.2 ± 1.5	MCI > HC	2	*p* < 0.05 cor
	HC 18	70.3 ± 4.7	10/8	28.3 ± 1.3	MCI < HC	0	
Wang et al. (2013)	MCI 18	73.7 ± 9.1	8/10	27.9 ± 1.2	MCI > HC	0	*p* < 0.05 uncor
	HC 16	70.7 ± 6.0	4/12	28.9 ± 0.9	MCI < HC	2	
Wang et al. (2011)	MCI 14	69.64 ± 6.88	8/6	26.64 ± 1.01	MCI > HC	7	*P* < 0.01 cor
	HC 14	68.07 ± 7.46	8/6	28.57 ± 0.65	MCI < HC	10	
Wang et al. (2012)	MCI 14	69.64 ± 6.88	8/6	26.64 ± 1.01	MCI > HC	0	*P* < 0.01 cor
	HC 14	68.07 ± 7.46	8/6	28.57 ± 0.65	MCI < HC	15	
**ReHo**							
Bai et al. (2008)	MCI 20	71.3 ± 3.8	10/10	27.2 ± 1.6	MCI > HC	0	*p* < 0.05 cor
	HC 20	69.4 ± 3.8	9/11	28.3 ± 1.4	MCI < HC	5	
Kang et al. (2018)	MCI 34	76.1 ± 4.7	7/27	22.7 ± 3.9	MCI > HC	0	*P* < 0.01 cor
	HC 38	74.0 ± 5.4	19/19	26.3 ± 2.3	MCI < HC	1	
Luo et al. (2018)	MCI 64	73.64 ± 4.76	34/30	27.75 ± 1.69	MCI > HC	1	*p* < 0.05 cor
	HC 49	73.33 ± 4.60	18/31	29.02 ± 1.20	MCI < HC	2	
Liu et al. (2021)	MCI 28	68.39 ± 4.65	14/14	/	MCI > HC	0	*P* < 0.01 cor
	HC 28	68.66 ± 5.09	14/14	/	MCI < HC	1	
Min et al. (2019)	MCI 10	69.80 ± 2.66	5/5	25.90 ± 0.74	MCI > HC	0	*p* < 0.05 uncor
	HC 10	69.90 ± 2.60	5/5	29.30 ± 0.82	MCI < HC	2	
Wang et al. (2015)	MCI 30	69.1 ± 5.8	12/18	26.2 ± 2.2	MCI > HC	1	*p* < 0.05 cor
	HC 32	70.1 ± 5.5	17/15	28.1 ± 1.5	MCI < HC	2	
Yuan et al. (2016)	MCI 36	66.8 ± 9.5	19/17	24.9 ± 3.4	MCI > HC	1	*P* < 0.001 cor
	HC 46	64.3 ± 7.8	27/19	28.5 ± 2.0	MCI < HC	2	
Zhang et al. (2021)	MCI 28	65.71 ± 6.895	13/15	27.07 ± 1.72	MCI > HC	4	*P* < 0.001 cor
	HC 37	63.86 ± 8.25	15/22	28.57 ± 1.21	MCI < HC	7	
**ALFF**							
Han et al. (2012b)	MCI 17	69.7 ± 7.6	10/7	25.2 ± 3.5	MCI > HC	1	*P* < 0.01 cor
	HC 18	66.5 ± 6.2	11/7	29.2 ± 0.7	MCI < HC	1	
Jia et al. (2015)	MCI 7	74.1 ± 7.8	2/6	27.0 ± 2.3	MCI > HC	1	*P* < 0.01 cor
	HC 15	70.2 ± 7.1	8/7	29.2 ± 1.3	MCI < HC	1	
Ren et al. (2017)	MCI 18	74.44 ± 10.6	8/10	/	MCI > HC	0	*P* < 0.01 uncor
	HC 21	71.71 ± 9.57	8/13	/	MCI < HC	1	
Zhao et al. (2014)	MCI20	65.11 ± 9.92	12/8	25.21 ± 2.24	MCI > HC	1	*p* < 0.05 cor
	HC 18	66.81 ± 7.43	10/8	29.31 ± 1.22	MCI < HC	1	

## Meta-analysis results

### Altered FC in MCI

**Figure 2:**
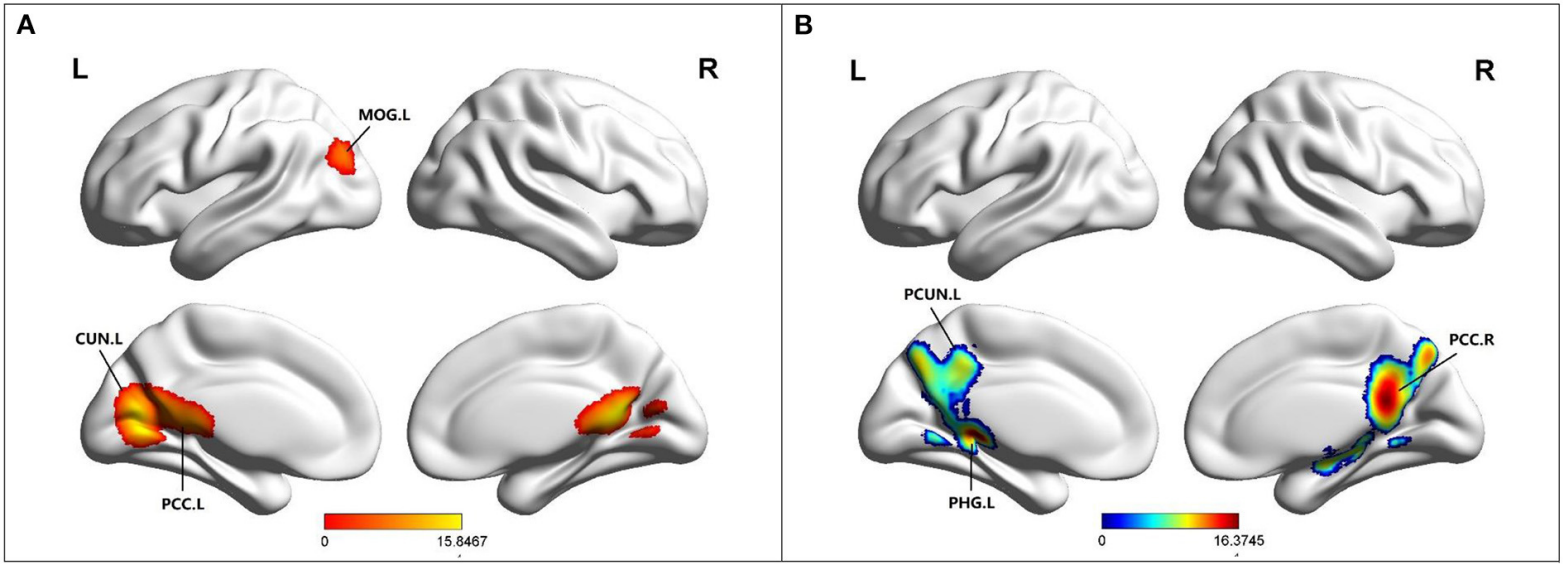
**(A)** Brain regions showing increased FC in MCI patients compared to HCs. **(B)** Brain regions showing decreased FC in MCI patients compared to HCs. MCI, amnestic mild cognitive impairment; HCs, healthy controls; FC, functional connectivity; MOG, middle occipital gyrus; CUN, cuneus; PCC, posterior cingulate; PCUN, precuneus; PHG, parahippocampal gyrus; R, right; L, left.

Compared to HC, MCI patients showed increased FC in the left posterior cingulate cortex (PCC), left cuneus (CUN), left middle occipital gyrus (MOG), and left culmen (Table 2; Figure 2). Besides, MCI patients revealed decreased FC in the left parahippocampal gyrus (PHG), bilateral posterior cingulate cortex (PCC), left precuneus (PCUN), bilateral culmen, right thalamus (THA), and right lentiform nucleus (Table 2; Figure 2).

**Table 2:** All clusters from the ALE analysis.

**Cluster**	**Volume(mm**3**)**	**MNI**			**Anatomical regions**	**Maximum ALE value**	**Side**	**BA**
		**X**	**Y**	**Z**				
**FC**								
**MCI** > **HC**								
1	40,608	−4	−64	10	Cuneus	0.002352157	Left	30
1	40,608	2	−56	12	Posterior Cingulate	0.002166562	Left	29
1	40,608	0	−52	2	Culmen	0.001069959	Left	/
1	40,608	−32	−78	26	Middle Occipital Gyrus	0.00102742	Left	19
**MCI**<**HC**								
1	56,064	−12	−36	2	Parahippocampal Gyrus	0.001835581	Left	27
1	56,064	8	−46	20	Posterior Cingulate	0.001813839	Right	30
1	56,064	2	−72	46	Precuneus	0.001593704	Left	7
1	56,064	−4	−44	40	Cingulate Gyrus	0.001404616	Left	31
1	56,064	−6	−56	4	Culmen	0.001283	Left	/
1	56,064	−2	−58	16	Posterior Cingulate	0.001112941	Left	29
1	56,064	28	−14	−12	Lentiform Nucleus	0.001041184	Right	/
1	56,064	−18	−58	26	Precuneus	0.001015452	Left	31
1	56,064	20	−32	2	Thalamus	0.00101406	Right	/
1	56,064	10	−12	−2	Thalamus	0.001005516	Right	/
1	56,064	10	−52	−4	Culmen	0.001000753	Right	/
**ReHo**								
**MCI** > **HC**								
1	52,072	−18	−76	44	Precuneus	0.000956739	Left	7
1	52,072	−24	−72	52	Superior Parietal Lobule	0.000956258	Left	7
1	52,072	−28	−82	42	Precuneus	0.000956017	Left	19
1	52,072	−12	−48	34	Precuneus	0.000949608	Left	31
2	20,232	0	−90	−3	Lingual Gyrus	0.000948747	Left	18
3	19,376	36	−33	−24	Parahippocampal Gyrus	0.000948747	Right	36
**MCI**<**HC**								
1	66,736	2	−66	40	Cuneus	0.001680949	Left	7
1	66,736	6	−48	16	Posterior Cingulate	0.001451564	Right	30
1	66,736	−4	−50	12	Posterior Cingulate	0.001185764	Left	29
1	66,736	−14	−98	4	Lingual Gyrus	0.001049166	Left	17
1	66,736	−4	−88	10	Cuneus	0.001045803	Left	17
1	66,736	14	−86	0	Lingual Gyrus	0.000990424	Right	18
1	66,736	8	−66	−6	Culmen	0.000956278	Right	/
1	66,736	−8	−72	−8	Culmen	0.00095379	Left	/
1	66,736	0	−52	2	Culmen	0.000610501	Left	/
**ALFF**								
**MCI** > **HC**								
1	45,608	−22	58	20	Superior Frontal Gyrus	0.000950644	Left	10
1	45,608	−36	58	−12	Middle Frontal Gyrus	0.000948823	Left	10
2	43,064	21	−44	−13	Culmen	0.000941543	Right	/
**MCI**<**HC**								
1	33,992	32	14	54	Middle Frontal Gyrus	0.000949095	Right	6
1	33,992	4	22	66	Superior Frontal Gyrus	0.000908387	Right	6
2	27,600	−3	45	28	Middle Frontal Gyrus	0.000934394	Left	9
3	23,176	60	−42	0	Middle Temporal Gyrus	0.000956006	Right	22
								

### Altered ALFF/fALFF in MCI

**Figure 3:**
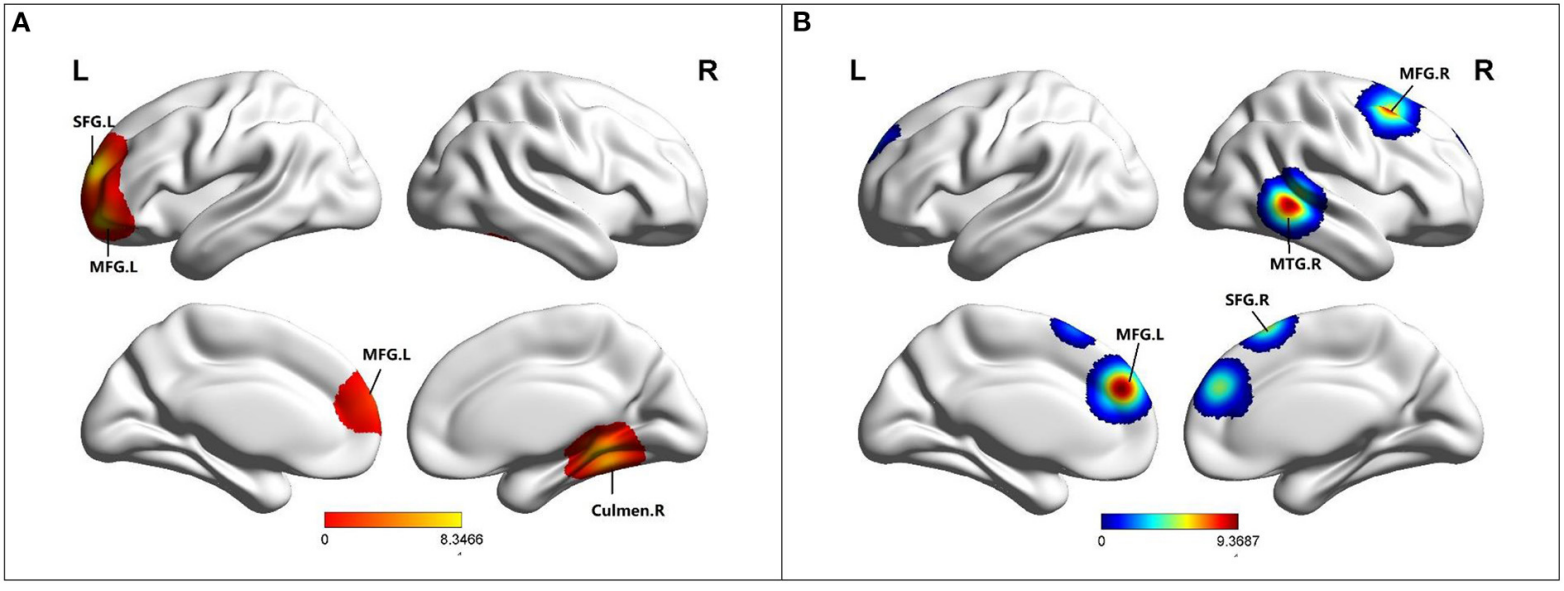
**(A)** Brain regions showing increased ALFF in MCI patients compared to HCs. **(B)** Brain regions showing decreased ALFF in MCI patients compared to HCs. MCI, amnestic mild cognitive impairment; HCs, healthy controls; ALFF, the amplitude of low-frequency fluctuation; SFG, superior frontal gyrus; MFG, middle frontal gyrus; MTG, middle temporal gyrus; R, right; L, left.

Compared to HC, MCI patients showed increased ALFF in the left middle frontal gyrus (MFG), right culmen, and left superior frontal gyrus (SFG) (Table 2; Figure 3). Besides, MCI patients revealed decreased ALFF in the bilateral middle frontal gyrus (MFG), right superior frontal gyrus (SFG), and right middle temporal gyrus (MTG) (Table 2; Figure 3).

### Altered ReHo in MCI

**Figure 4:**
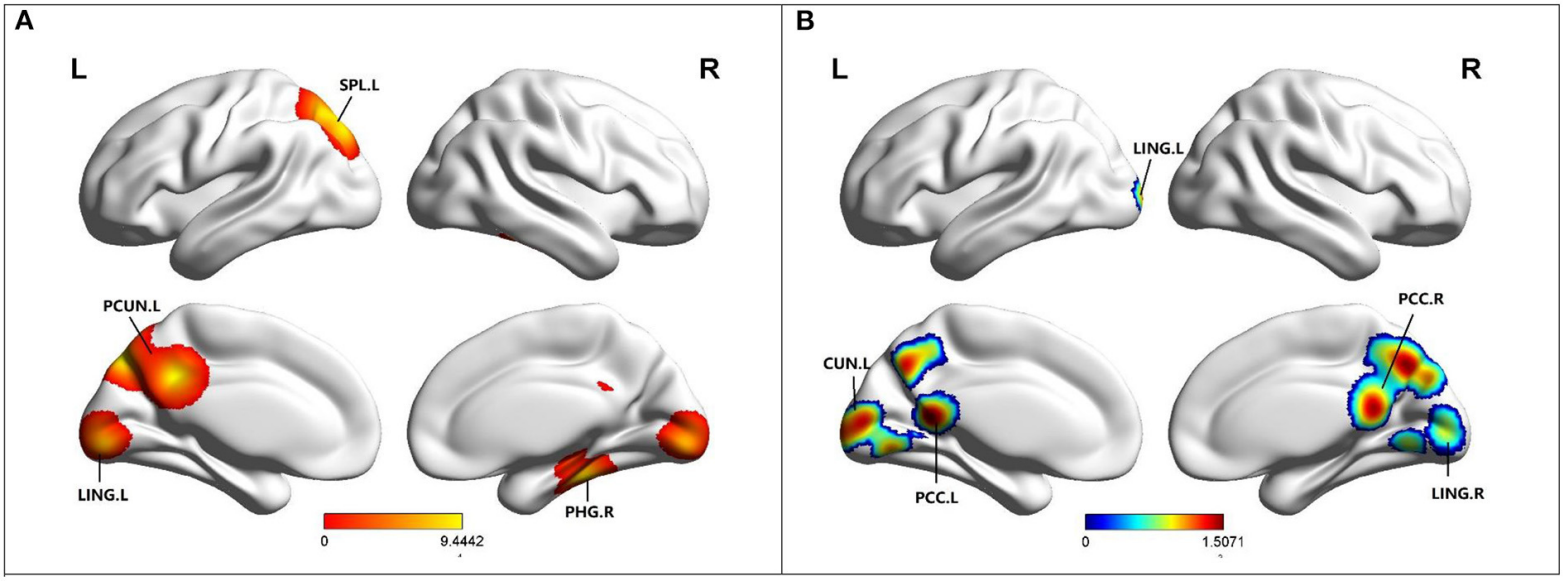
**(A)** Brain regions showing increased ReHo in MCI patients compared to HCs. **(B)** Brain regions showing decreased ReHo in MCI patients compared to HCs. MCI, amnestic mild cognitive impairment; HCs, healthy controls; ReHo, regional homogeneity; PCUN, precuneus; PCC, posterior cingulate; PHG, parahippocampal gyrus; LING, lingual gyrus; SPL, superior parietal lobule; R, right; L, left.

Compared to HC, MCI patients showed increased ReHo in the left precuneus (PCUN), left superior parietal lobule (SPL), left lingual gyrus (LING), and right parahippocampal gyrus (PHG) (Table 2; Figure 4). Besides, MCI patients revealed decreased ReHo in the left cuneus (CUN), bilateral posterior cingulate cortex (PCC), bilateral lingual gyrus (LING), and bilateral culmen (Table 2; Figure 4).

## Discussion

In previous studies, there have been many studies on the activation state of episodic memory, summarizing many brain areas related to episodic memory. But as far as we know, this is the first meta-analysis to study functional changes in the relevant brain regions in the resting state. Compared with the healthy group, the specific abnormality of episodic memory brain regions in MCI were mainly located in the frontal lobe, including the superior frontal gyrus and middle frontal gyrus; the limbic lobe, including posterior cingulate and parahippocampal gyrus; the occipital lobe, including cuneus, lingual gyrus, and middle occipital gyrus; the temporal lobe, including middle temporal gyrus; the parietal lobe, including precuneus; and sub-lobar, including the thalamus and lentiform nucleus. Overall, the specific functional changes in brain regions analyzed in this paper are meaningful and may help to search for radiologic biomarkers for the course of MCI disease.

### Specific imaging abnormal changes

In our study, the brain regions with increased FC were mainly located in the left CUN, the left MOG and the left PCC. Episodic memory is the ability to remember, recall, and encode information about events and experiences regarding a specific time and place (Bai et al., 2016). Located in the occipital lobe, the cuneus, LING, and MOG are not only involved in visual-spatial processing, but also play important roles in memory formation, object and face recognition, and perception of distance and depth (Chen et al., 2020; Li et al., 2020). Thus, damage to the cuneus and MOG leads to impaired episodic memory formation and thus impaired episodic memory function in MCI patients. The results of this study indicate that the increase of FC in the cuneus and MOG may compensate for this functional defect. Another important brain region is the left PCC. The PCC is a highly connected and metabolically active brain region (Leech and Sharp, 2014). Histopathological (Rowe et al., 2007) and structural (Shiino et al., 2006) and functional imaging (Liang et al., 2008) studies consistently demonstrated that PCC plays an important role in the pathogenesis of AD and MCI. Bai et al. (2009b) found that PCC connectivity with diffuse frontal cortex and ITG increased in MCI patients. FC often respond to functional characteristics by revealing whether there is disruption or compensation in connectivity between brain regions (Liu et al., 2020). Thus, the increase of FC in PCC contributes to the relatively stable level of episodic memory function in MCI patients over a period of time.

Our study also observed decrease FC in the left PHG, bilateral PCC, left precuneus, right thalamus, and right lentiform nucleus. Studies showed that the earliest brain changes in MCI, such as volume loss, occur in the HIP and PHG, located in the medial temporal lobe (MTL) (Masdeu et al., 2005). MTL is known to be one of the key brain regions for episodic memory, and the function of the PHG is thought to be involved in the conversion of information temporarily present in the HIP into more permanent storage in cortical associated regions (Powell et al., 2004). As a result, FC in the PHG decreases, and connections to other brain regions are reduced, leading to impaired long-term storage of episodic memories. Changes in the precuneus begin 10 to 20 years before clinical symptoms of dementia appear (Bateman et al., 2012) and FC decreased in precuneus is a high-risk factor for AD (Tao et al., 2017). The reason may be that the precuneus often shows high levels of amyloid deposition and is susceptible to structural and functional changes in MCI (Tao et al., 2017). One study showed that FC in the precuneus was significantly reduced in MCI patients after removing the effect of cortical thickness. This is consistent with the research results of this study. Resting-state studies showed that there is a strong functional connection between the thalamus and the precuneus under normal conditions (Tomasi and Volkow, 2010). In this study, FC decreased in both the thalamus and precuneus. Therefore, these results suggest that the important functional connection between the thalamus and the precuneus have been damaged and is in the stage of decompensation.

ALFF measures the total power of a given time process over a specific frequency range, and reflects the level of glucose metabolism in the brain region (Yu-Feng et al., 2007; Zhang et al., 2020). ALFF is an indicator that can quantify local spontaneous neuronal activity. The results of this study showed that ALFF of the left SFG and MFG increased while that of the right SFG and bilateral MFG decreased. A study showed that patients with MCI have reduced frontal gray matter volume (Han et al., 2012a), and gray matter loss and regional brain atrophy may lead to ALFF decrease (Zhao et al., 2014). The frontal cortex is an important region involved in-memory processing. Subjects with higher bilateral frontal cortex activity can perform semantic memory and episodic memory tasks better (Zhao et al., 2014). It has been proposed that MCI patients may have a frontal cortex compensation network, whose increased activity may be used to compensate for the dysfunction of the medial temporal lobe (Grady et al., 2005). The compensation mechanism of the frontal lobe can make patients maintain a relatively balanced low cognitive state in a period. In this study, there was both an increase and decrease in ALFF in the frontal lobe, suggesting that compensation and damage in the frontal lobe may occur at different stages of MCI, leading to such results. Therefore, the ALFF results in this study reflect the physiological state of the brain and provide a new understanding of the pathogenesis and diagnosis of MCI.

ReHo can effectively evaluate resting-state brain activity. Based on the hypothesis that brain activity is more likely to occur in clusters rather than in a single voxel, ReHo is calculated using Kendall's coefficient of concordance (KCC), which evaluates the similarity between the time series of a given voxel and its nearest neighbors (Zang et al., 2004). It reflects local consistency of spontaneous brain activity and can sensitively detect FC of abnormal brain regions (Yuan et al., 2016). ReHo increased in the left precuneus, right PHG and left LING, while decreased in the left cuneus, bilateral PCC and bilateral LING. Precuneus can be activated in different tasks, such as episodic memory, visual-spatial imagery and self-processing (Cavanna and Trimble, 2006). Multiple synaptic connections between the PHG and HIP provide the structural basis for memory processing (Powell et al., 2004). The PCC is an important brain region for episodic memory. According to the results of this study, we can speculate that the function of the PCC is generally declining despite a small amount of functional compensation. In addition to visual formation, LING and cuneiform are also involved in the formation of episodic memory. The ReHo of the left LING increased while that of the bilateral LING decreased, reflecting the difference in spontaneous activity of the LING. These results suggest that during the MCI stage, LING may be susceptible to cognitive impairment and undergo different functional changes.

### Interactive brain networks

Through the above results and analysis, it is found that episodic memory is not limited to one brain region. This is consistent with previous research showing that episodic memory is a physiological activity formed through an interconnected brain network (Gliebus, 2018). This study shows that episodic memory brain regions overlap with default mode network, salience network and executive control network. At the same time, other studies showed that cognitive functions such as episodic memory depend on interactions between these brain networks (Gliebus, 2018). Therefore, we can speculate that DMN, ECN and SN may participate in the complex physiological activities of episodic memory through some specific brain regions.

DMN is associated with internally-directed cognition and is involved in neutralizing cognitive and emotional processing, monitoring the surrounding environment, and more importantly, episodic memory (Krajcovicova et al., 2017). In this study, the brain regions overlapped with DMN are precuneus, PCC, PHG and HIP. The precuneus and PCC are activated during episodic memory (Cavanna and Trimble, 2006). Studies showed that reduced function in the precuneus and PCC may lead to disruptions in brain networks responsible for maintaining normal cognitive function, leading to the development of AD (Yokoi et al., 2018). The HIP and PHG gyrus, located in the medial temporal lobe, plays an important role in connecting information from different cortical regions. Moreover, damage to the MTL can affect the formation of new memories, information retains and retrieval (Gliebus, 2018). Hence, DMN is involved in episodic memory through the above brain regions, and changes in functional indicators of these brain regions can be used as markers of impaired episodic memory and MCI.

ECN mainly includes the lateral posterior parietal cortex and dorsolateral prefrontal cortex (DLPFC), which are activated during higher cognitive functions and responsible for a wide range of executive functions (Liang et al., 2016). The results of this study involve functional changes in the frontal lobe. There is evidence that DLPFC can help memory formation by making connections between different items (Blumenfeld et al., 2011). Wang et al. 's meta-analysis showed that in MCI, the right DLPFC activity was higher, compensating for the low activity of the left DLPFC and MTL encoding the plot information (Wang et al., 2016). In addition, episodic memory requires ECN attention to allocate, guide, control, organize and monitor information during encoding and retrieval (Gliebus, 2018). These results indicate that ECN can participate in the physiological function of episodic memory through both brain regions and the whole network function.

SN can recognize stimuli, guide behavioral responses, and play a major role in emotion, attention, and perception (Menon, 2011; Uddin, 2015). Specifically, when salient events occur, SN activates brain networks and directs DMN and ECN to respond appropriately to stimuli (Menon, 2011). Although there is no clear evidence in this paper that the anterior cingulate gyrus (ACC), one of the core brain regions of SN, is involved in episodic memory, it constitutes the cingulate gyrus together with PCC, an important brain region of episodic memory. This study showed that the function of PCC decreased. However, Song et al. (2021) founded that ACC FC was elevated in MCI patients. According to the theory of aging, the forebrain compensates for the damage done by the back brain (Skouras et al., 2019). We can speculate that ACC is involved in the episodic memory of MCI patients in the form of compensation mechanism, that is, SN can indirectly participate in episodic memory through ACC.

## Clinical implications

Our article is a quantitative neuroimaging meta-analysis based on a comprehensive literature search summarizing the results of individual studies available on MCI. Our findings suggest that episodic memory-related brain regions in MCI patients exhibit specific functional changes, which provide new ideas and entry points for disease monitoring, process development, and treatment evaluation. Moreover, these related brain regions, such as the precuneus, parahippocampal gyrus, posterior cingulate gyrus, and cuneiform, provide the basis for therapeutic targets for some specific treatments such as transcranial direct current stimulation and deep brain stimulation. In conclusion, our study strongly demonstrates the imaging features of damaged brain regions in MCI patients, laying a foundation for further research on the pathogenesis of the disease and providing new insights for the treatment of the disease.

## Limitation

There are some limitations to this meta-analysis. The first is the heterogeneity of age, gender, and disease severity of the subjects included in the literature, and this heterogeneity cannot be assessed by the ALE calculation. So, this may affect our research results. Second, due to the limited literature included, MCI patients are not divided into aMCI and naMCI, and there is no control for single or multiple dysfunctions, early or late MCI, etc. Third, this meta-analysis included the results of most of the major brain regions related to episodic memory, but a few brain regions may not be included. Finally, unavailable key data or non-English publications are excluded, which may lead to incomplete data.

## Conclusions

We performed three ALE analyses, including FC, ALFF, and ReHo, to determine the functional imaging findings of episodic memory-related brain regions in MCI patients. The results showed that the related brain regions were concentrated in the precuneus, parahippocampal gyrus, posterior cingulate gyrus, and cuneus with characteristic changes. In addition, the relationship between brain regions and brain networks related to episodic memory is also discussed. The specific changes in these brain regions can help identify the potential imaging markers of MCI, deepen the understanding of MCI, and provide new directions for diagnosis, treatment, and evaluation of treatment efficacy.

## Data availability statement

The original contributions presented in the study are included in the article/supplementary material, further inquiries can be directed to the corresponding author.

## Author contributions

XL, QY, CXu, JC, CXi, and WQ designed the study. HG, ZY, SC, YS, and HW helped with literature search, data extraction, and data analysis. XL, QY, and CXu were performed meta-analysis and drafting of the manuscript. All authors checked the data of the article, revised the content of the article, and finally agreed to the upcoming version.

## Funding

This study was supported by the National Natural Science Foundation of China (No. 81701675), the Key Project supported by Medical Science and technology development Foundation, Nanjing Department of Health (No. JQX18005), the Key Research and Development Plan (Social Development) Project of Jiangsu Province (No. BE2018608), and Jiangsu Provincial Natural Science Foundation-Youth Foundation Projects (BK20180370).

## Conflict of interest

The authors declare that the research was conducted in the absence of any commercial or financial relationships that could be construed as a potential conflict of interest. The handling editor Y-CC declared a shared parent affiliation with the author(s) at the time of review.

## Publisher's note

All claims expressed in this article are solely those of the authors and do not necessarily represent those of their affiliated organizations, or those of the publisher, the editors and the reviewers. Any product that may be evaluated in this article, or claim that may be made by its manufacturer, is not guaranteed or endorsed by the publisher.
